# Successful Pregnancy Management of a Woman With Severe Methylmalonic Acidemia

**DOI:** 10.1002/jmd2.70009

**Published:** 2025-05-12

**Authors:** M. Woidy, K. Tsiakas, M. Mahmud, G. Eich, S. Loos, J. Lisfeld, S. Schultz, A. C. Tallarek, K. Hecher, T. B. Huber, A. C. Muntau, G. Gramer

**Affiliations:** ^1^ Department for Inborn Metabolic Diseases University Children's Hospital, University Medical Center Hamburg‐Eppendorf Hamburg Germany; ^2^ University Children's Hospital, University Medical Center Hamburg‐Eppendorf Hamburg Germany; ^3^ III.Department of Medicine University Medical Center Hamburg‐Eppendorf Hamburg Germany; ^4^ Hamburg Center for Kidney Health (HCKH), University Medical Center Hamburg‐Eppendorf Hamburg Germany; ^5^ Institute of Human Genetics, University Medical Center Hamburg‐Eppendorf Hamburg Germany; ^6^ Department of Obstetrics and Fetal Medicine University Medical Center Hamburg‐Eppendorf Hamburg Germany

**Keywords:** chronic kidney disease, interdisciplinary management, pregnancy, severe methylmalonic acidemia

## Abstract

Isolated methylmalonic acidemia (MMA) is a rare, genetically heterogeneous group of metabolic disorders resulting from a deficiency of the enzyme methylmalonyl‐CoA mutase (MMUT), defects in the metabolism of its cofactor, adenosylcobalamin, or deficiency of the enzyme methylmalonyl‐CoA epimerase. With improved awareness, earlier diagnosis, and advances in care, women with MMA are increasingly reaching childbearing age, and successful pregnancies have been documented in patients with milder forms of the disease. This report details, for the first time, the management and outcomes of pregnancy in a woman with severe mut^0^ deficiency and concomitant advanced chronic kidney disease (CKD) progressing to end‐stage renal disease (ESRD) requiring initiation of hemodialysis at 21 weeks' gestation. At 20 weeks, fetal ultrasound revealed fetal growth restriction (FGR), necessitating close monitoring and dietary adjustments to meet the patient's increased nutritional needs. Despite these challenges, she remained metabolically stable until delivery. At 35 weeks, she delivered a 1.64 kg male SGA newborn via cesarean section. The newborn presented with mild retrognathia, a soft palate cleft, mild hypospadias, mild ventriculomegaly, and hypoplasia of the corpus callosum and cerebellum without the need for immediate intervention. The mother experienced a mild metabolic decompensation on the fifth postpartum day, which was promptly managed by additional renal replacement therapy. At 3 months postpartum, both mother and child were doing well, with no further metabolic complications observed. This case report demonstrates that pregnancy in patients with severe mut^0^ deficiency is challenging and requires a close interdisciplinary management but can be carried out with a favorable outcome.

**Summary**

- We present to our knowledge the first documented case of a successful pregnancy in a woman with severe mut^0^ deficiency and concomitant advanced chronic kidney disease.
- The patient required hemodialysis during pregnancy.
- This case underscores the critical importance of close interdisciplinary management involving experts in nephrology, obstetrics, genetics, metabolic medicine, and nutritional counseling.

## 1 Introduction

Isolated methylmalonic acidemia (MMA) is a rare group of inborn errors of metabolism caused either by a deficiency of the enzyme methylmalonyl‐CoA mutase (MMUT), defects in the transport or metabolism of its cofactor, adenosylcobalamin, or deficiency of the enzyme methylmalonyl‐CoA epimerase [1]. MMUT catalyzes the conversion of methylmalonyl‐CoA to succinyl‐CoA in the propionate pathway. A deficiency in this enzyme leads to the accumulation of methylmalonyl‐CoA and propionyl‐CoA, which are subsequently converted to their corresponding acids, methylmalonic acid and propionic acid. This accumulation, along with the buildup of other toxic metabolites such as 2‐methylcitrate and additional factors that are not yet fully understood, contributes to the disease's multi‐organ manifestations. These include abnormalities in the central nervous system, optic atrophy, sensorineural hearing loss, recurrent episodes of pancreatitis, arrhythmias and/or cardiomyopathy, and tubulointerstitial nephritis with progressive impairment of renal function [1]. Patients with a complete absence of enzyme activity (mut^0^) typically present within the first days of life with acute metabolic deterioration with ketoacidosis and hyperammonemia, whereas patients with low to moderate residual activity (mut^−^) may exhibit symptoms later in life. This also applies to defects in the metabolism of the cofactor adenosylcobalamin. Where available, newborn screening for MMA has the potential to detect especially the attenuated (mut‐) forms prior to the onset of symptoms [1].

Due to earlier diagnosis and improved care, including the option of liver and/or kidney transplantation, women with MMA are more frequently reaching childbearing age [2]. Seventeen pregnancies have been reported in women with varying degrees of MMA severity [2]. In general, severe metabolic crises were not observed, and the outcomes for both mother and child were favorable [2]. However, one child born to a mother with MMA experienced fetal growth restriction (FGR), which was attributed to inadequate nutritional intake [3]. Another pregnant woman, identified as mut^−^, experienced a 30% decline in renal function during the second trimester, despite the absence of related symptoms such as hypertensive episodes [4]. To our knowledge, all women with successful pregnancies reported in the literature had attenuated forms of MMA (mut^−^), and none had the most severe form of MMA. Here, we describe the interdisciplinary management and outcome of a pregnant woman with severe mut^0^ deficiency.

## 2 Case Study

**FIGURE 1:**
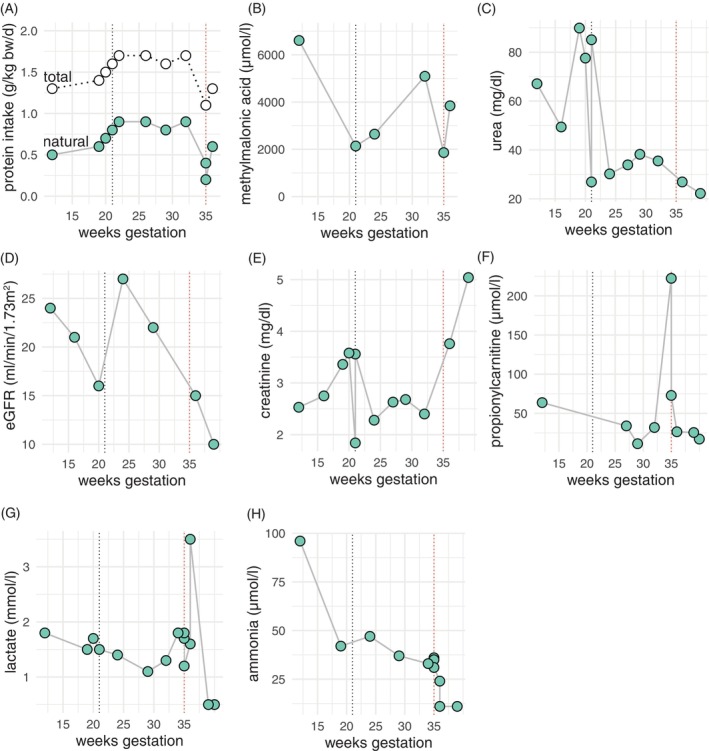
Laboratory parameters of the mother by gestational age. Each panel represents the course of the respective laboratory parameter as indicated on the *y*‐axis by weeks' gestation. The black dashed line indicates initiation of hemodialysis. The red dashed line marks the timepoint of birth by caesarian section.

A 35‐year‐old woman with genetically confirmed mut^0^ deficiency (homozygous pathogenic variant in *MMUT* (c.1106G>A, p.R369H), previously characterized as mut^0^ [5]) was admitted for the first time to our metabolic outpatient clinic for further evaluation and management of her pregnancy. At the time of presentation, she was in her 12th week of gestation. Her treatment included a low‐protein diet with 0.5 g/kg bw/day natural protein and 1.3 g/kg bw/day total protein intake (Figure 1A). She had a percutaneous endoscopic gastrostomy (PEG) tube for amino acid supplementation and medication administration. She had been diagnosed with MMA at 14 months of age following a severe metabolic decompensation, which required intensive medical care. At the age of 14 years, she was offered organ transplantation due to metabolic instability, which she and her parents declined. Since then, she has experienced recurrent metabolic decompensations, was diagnosed with a learning disability, and developed optic atrophy at the age of 21 years. At the age of 31 years, she experienced a severe metabolic decompensation with NSTEMI, acute kidney insufficiency, and fluid overload necessitating hemodialysis. Thereafter, the patient developed chronic kidney disease (CKD) that was treated with conservative management and only during metabolic decompensations with dialysis. The patient's last echocardiographic examination in our clinic showed normal biventricular function but mild mitral‐valve insufficiency and mild left‐ventricular hypertrophy.

At the time of her initial evaluation at our clinic, her plasma methylmalonic acid levels (6606 μmol/L) and urine levels (1165.5 μmol/mmol creatinine) were significantly elevated (Figure 1B), and her kidney function was severely compromised (serum creatinine 2.53 mg/dL, eGFR 24 mL/min/1.73m^2^, cystatin C 2.76 mg/L, cystatin GFR 21 mL/min) with high blood urea nitrogen (BUN) levels, metabolic acidosis, and electrolyte imbalances such as hyperkalemia and hyperphosphatemia (Figure 1C–E). To address the acidosis and correct the electrolyte disturbances, her diet was adjusted to reduce potassium and phosphate intake, and her medication, including oral sodium bicarbonate supplementation, was modified accordingly. The combination of severe iron deficiency and renal anemia was managed through intravenous iron supplementation and the initiation of an erythropoiesis‐stimulating agent.

With progressing pregnancy, we expected further deterioration of kidney function [4] and an associated increased risk for preeclampsia and metabolic disturbances due to azotemia with risks for the patient and the unborn child. Consequently, we engaged a multidisciplinary team (nephrology, obstetrics, metabolic medicine, genetics, nutrition counseling) and offered a thorough counseling to the patient and her family regarding the increased risk for iatrogenic preterm birth and the need for hemodialysis. An abortion based on maternal indication was not an option for the patient as she wished to continue the pregnancy. Although the patient initially refused hemodialysis due to concerns about a lifelong dependence on dialysis, with deteriorating kidney function (serum creatinine 3.56 mg/dL, eGFR 14 mL/min/1.73m^2^, BUN 77 mg/dL) and associated complications such as sustained severe metabolic acidosis despite substitution and hypervolemia she eventually agreed, and after the insertion of a tunneled central venous catheter, hemodialysis was initiated at 21 weeks of gestation. Hemodialysis was performed for 5–6 h, 6 days per week, to optimize pregnancy outcomes for both mother and child, while preventing hypertensive episodes and metabolic complications associated with CKD and MMA.

By 20 weeks of gestation, ultrasound examination revealed FGR and signs of mild intracranial abnormalities, including bilateral mild ventriculomegaly, cerebellar hypoplasia, and mild hypoplasia of the corpus callosum. To investigate potential genetic causes, amniocentesis was performed. Chromosome analysis was normal, and trio exome sequencing did not reveal pathogenic variants in known disease genes in the fetus.

Regarding preexisting diseases in the patient's partner, he has a history of congenital heart disease (dextro‐transposition of the great arteries) requiring heart transplantation, learning disability, and unilateral limb spasticity. Chromosome analysis, in addition to exome sequencing, was unremarkable in the father; he especially had no pathogenic variants in the *MMUT* gene, indicating no increased risk of MMA for the child.

To meet the increasing nutritional demands of advancing pregnancy and daily hemodialysis, the patient's amino acid and nutrient levels were closely monitored, and her natural protein intake was gradually increased to 0.9 g/kg bw/day, resulting in a total protein intake of 1.7 g/kg bw/day (0.8 g/kg bw/day from amino acid substitutes) (Figure 1A). Despite this increase, her metabolic parameters remained stable throughout the pregnancy, and no episodes of metabolic decompensation were observed (Figure 1B,F–H). Moreover, methylmalonic acid levels decreased during dialysis (Figure 1B). Due to hypervolemia with consecutive hypertensive blood pressure, the patient was admitted to our inpatient obstetric clinic at 29 weeks of gestation for intensified feto‐maternal monitoring.

**FIGURE 2:**
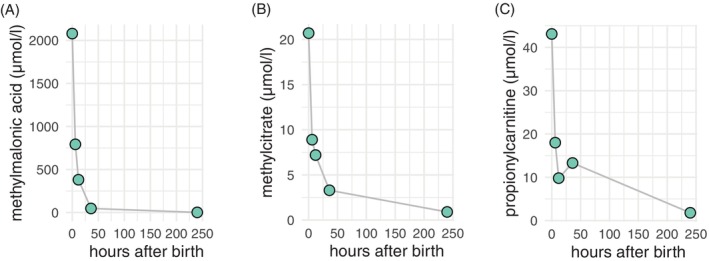
Postnatal metabolic parameters in the newborn. Methylmalonic acid, methylcitrate and propionylcarnitine levels as measured in dried blood spots at different time points after birth are depicted.

Despite ongoing hemodialysis and adjusted medication, the patient's blood pressure gradually rose (up to 180/120 mmHg), and ultrasound showed FGR with restricted blood flow to the umbilical cord and signs of cerebral blood‐flow redistribution. At 35 weeks of gestation, a male newborn was delivered via cesarean section. He weighed 1640 g (5th percentile, −1.65z), measured 39.5 cm (1st percentile, −2.31z) in length, had a head circumference of 29 cm (3rd percentile, −1.88z), and received Apgar scores of 8/9/9. He was admitted to the neonatal intensive care unit. Initial tests showed significantly elevated levels of methylmalonic acid, methylcitrate, and propionylcarnitine in the newborn, but these normalized within a few days without any intervention (Figure 2). A cerebral ultrasound confirmed mild ventriculomegaly, hypoplasia of the corpus callosum, and hypoplasia of the cerebellum. Additionally, the newborn was found to have mild retrognathia, a soft palate cleft, and mild hypospadias, none of which required immediate intervention.

At the mother's request, the newborn was fed with regular formula instead of breast milk, and by the time of discharge from the hospital, his weight had increased to 2296 g. At 3 months after birth, due to the soft palate cleft, the infant was fed by a Haberman Feeder and weighed 3670 g. At that time, he was developing well on physio‐ and logotherapy but showed mild plagiocephaly.

Peripartum management of the mother included intravenous infusions of glucose (3 g/kg bw/day) and carnitine (100 mg/kg bw/day), along with close monitoring of her acid–base balance, blood lactate, and ammonia levels. Dietary protein intake was paused for 24 h and gradually reintroduced, starting with 0.2 g/kg bw/day of natural protein and 0.9 g/kg bw/day from an amino acid mixture on the second day postpartum. Protein intake was progressively increased, and the glucose infusion was discontinued by the third day.

On the fifth day, the mother developed metabolic acidosis with mildly elevated blood lactate levels and hyperkalemia. For continuous monitoring and therapy, she was subsequently admitted to the intensive care unit, and continuous renal replacement therapy for a period of less than 24 h was initiated. Shortly thereafter, no further episodes of metabolic decompensation occurred, and intermittent hemodialysis was continued. Three months after delivery, the mother did not experience any further metabolic decompensation. She reported difficulties managing her child and her diet, which often results in reduced nutritional intake. She is still on hemodialysis 3 days per week, and metabolic parameters stayed stable (MMA level in urine 904 μmol/L, propionylcarnitine in DBS 24.11 μmol/L, ammonia 53 μmol/L).

## 3 Discussion

As a result of earlier diagnosis, increased awareness, and improved medical care, more women with inborn errors of metabolism are reaching childbearing age [6]. MMA is one such inborn error of metabolism, characterized by a highly heterogeneous clinical presentation. Seventeen pregnancies in women with mild MMA have been reported, all with favorable outcomes for both mother and child [2]. No metabolic decompensation was observed, and only one case involved FGR [2]. However, none of the reported cases suffered from such a severe mut^0^ deficiency as in our case.

The fetus in our case showed severe FGR, first detected at 20 weeks of gestation. At that time, the mother's CKD was poorly managed due to her initial refusal of hemodialysis, resulting in sustained chronic metabolic acidosis and elevated BUN levels. These factors likely contributed negatively to fetal growth. Indeed, high urea levels have been linked to an increased risk of FGR [7].

Other factors may also have influenced fetal development. First, our patient followed a protein‐restricted diet, which we closely monitored and gradually adjusted based on her nutritional and metabolic status. Second, until daily hemodialysis was initiated, toxic substances, such as methylmalonic acid, were extremely elevated in the mother's blood. In our patient, methylmalonic acid levels slightly decreased during dialysis as previously reported [8]. This could be significant, as methylmalonic acid is known to passively diffuse across the placenta and may accumulate in the fetus [9]. We found high levels of methylmalonic acid, methylcitrate, and propionylcarnitine in the newborn—almost as high as those in the mother—immediately after birth (Figure 2). It can be assumed that the fetus was exposed to even higher concentrations during the first two trimesters, given the mother's poor metabolic control. It is unclear to what extent the fetal metabolism can manage this exposure.

Moreover, in addition to FGR, we observed other clinical features in the newborn that suggest disturbances in early embryogenesis, such as hypospadias, soft cleft palate, hypoplasia of the corpus callosum and cerebellum, as well as retrognathia. Genetic diagnostics including chromosome and trio exome analysis did not reveal an underlying genetic cause. While we cannot confirm or rule out the potential negative effects of elevated maternal metabolites on fetal development during early pregnancy, it is theoretically possible that metabolite concentrations could reach levels capable of disrupting cellular energy metabolism, potentially through a recently proposed aberrant post‐translational modification known as increased methylmalonylation [10]. Mitochondria play a crucial role in placentation and fetal development [11], and it is possible that disturbed mitochondrial function contributed to the observed growth restriction and mild abnormalities in the infant. To our knowledge, toxic effects of methylmalonic acid on the fetus have not been previously described [2]. However, none of the published cases reported methylmalonic acid levels as consistently elevated throughout pregnancy as those seen in our patient.

In our patient, the pregnancy was complicated by severe kidney dysfunction, necessitating hemodialysis started at 21 weeks of gestation. Fluid overload and elevated maternal blood pressure ultimately led to a preterm cesarean section at 35 weeks. The postpartum period is a highly catabolic state and represents a critical phase for patients with inborn errors of metabolism [6]. Despite carefully reintroducing dietary protein, our patient experienced mild metabolic decompensation, likely due to the stress of delivery complicated by acute anemia afterward and the postpartum readjustment to the new metabolic situation, which required acute hemodialysis on the fifth day postpartum. Apart from this episode, the pregnancy was metabolically stable, with no episodes of lactic acidosis or hyperammonemia observed.

To conclude, we here describe to our knowledge the first case of successful pregnancy management in a woman with severe mut^0^ deficiency and concomitant advanced CKD requiring hemodialysis. This case highlights the importance of a close interdisciplinary management including experts from nephrology, obstetrics, genetics, metabolic medicine, and nutrition counseling.

## Author Contributions

M.W. and G.G. conceived the case study and are responsible for the overall integrity of the content of the manuscript. M.W. wrote the initial draft of the manuscript and collected clinical data. All authors were involved in the patient's care and reviewed the final manuscript.

## Consent

Written consent was obtained from the patient.

## Conflicts of Interest

G.E. reports grants and personal fees from Bioporto, outside the submitted work. The other authors declare no conflicts of interest.

## Data Availability

The data that support the findings of this study are available from the corresponding author upon reasonable request.
